# A 4-year study of bovine reproductive hormones that are induced by pharmaceuticals and appear as steroid estrogenic pollutants in the resulting slurry, using in vitro and instrumental analytical methods

**DOI:** 10.1007/s11356-023-31126-y

**Published:** 2023-11-25

**Authors:** Eduárd Gubó, Judit Plutzer, Tibor Molnár, Dóra Pordán-Háber, Lili Szabó, Zoltán Szalai, Richard Gubó, Pál Szakál, Tamás Szakál, László Környei, Ákos Bede-Fazekas, Renátó Kalocsai

**Affiliations:** 1 Albert Kázmér Faculty, Széchenyi István University, Vár Tér 2, 9200 Mosonmagyaróvár, Hungary; 2 reAgro Research and Development Ltd., Győrújbarát, Hungary; 3 Research Centre for Astronomy and Earth Sciences, Hungarian Academy of Sciences, Geographical Institute, Budapest, Hungary; 4 SynCat@Beijing, Synfuels China Technology Co. Ltd., Leyuan South Street II, No.1, Huairou District, Beijing, 101407 China; 5 National Energy Center for Coal to Liquids, Synfuels China Co., Ltd., Beijing, 101400 China; 6 Department of Mathematics and Computational Sciences, Széchenyi István University, Győr, Hungary; 7 Department of Environmental and Landscape Geography, Eötvös Lóránd University, Budapest, Hungary

**Keywords:** Slurry, Manure, Endocrine disrupting chemicals (EDCs), Steroidal estrogens, Xenoestrogens, *Oestrus* induction

## Abstract

The main objective of the research was to study the environmental “price” of the large-scale, milk production from a rarely known perspective, from the mapping of the estrogenic footprint (the amount of *oestrus-*inducer hormonal products, and the generated endoestrogens) in the resulting slurry in a dairy cow farm. These micropollutants are endocrine-disrupting chemicals (EDCs) and can be dangerous to the normal reproductive functions even at ng/kg concentration. One of them, 17ß-estradiol, has a 20,000 times stronger estrogenic effect than bisphenol-A, a widely known EDC of industrial origin. While most studies on EDCs are short-term and/or laboratory based, this study is longitudinal and field-based. We sampled the slurry pool on a quarterly basis between 2017 and 2020. Our purpose was testing the estrogenic effects using a dual approach. As an effect-based, holistic method, we developed and used the YES (yeast estrogen screen) test employing the genetically modified *Saccharomyces cerevisiae* BJ3505 strain which contains human estrogenic receptor. For testing exact molecules, UHPLC-FLD was used. Our study points out that slurry contains a growing amount of EDCs with the risk of penetrating into the soil, crops and the food chain. Considering the Green Chemistry concept, the most benign ways to prevent of the pollution of the slurry is choosing appropriate *oestrus*-inducing veterinary pharmaceuticals (OIVPs) and the separation of the solid and liquid parts with adequate treatment methods. To our knowledge, this is the first paper on the adaptation of the YES test for medicine and slurry samples, extending its applicability. The adapted YES test turned out to be a sensitive, robust and reliable method for testing samples with potential estrogenic effect. Our dual approach was successful in evaluating the estrogenic effect of the slurry samples.

**Graphical Abstract:**

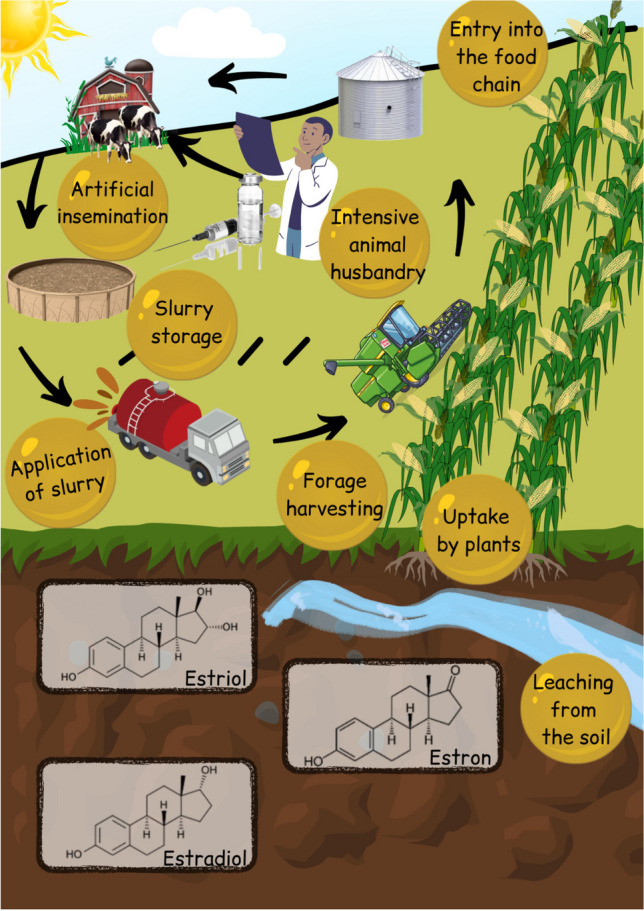

**Supplementary Information:**

The online version contains supplementary material available at 10.1007/s11356-023-31126-y.

## Introduction

Green Chemistry is mainly about how to protect the environment and ourselves from the adverse effects of our own chemicals and how to convert harmful materials to more benign ones. Slurry is once thought to be an inherently safe and benign source of nutrients. Due to the intensifying technologies of modern agriculture (such as the increasing use of *oestrus*-inducer hormonal products, other pharmaceuticals), however, slurry ends up in the environment as a carrier of endocrine-disrupting chemicals (EDCs), causing growing concerns (Li et al. 2020). These micropollutants (MPs) can be harmful to living creatures even at extremely low concentrations (1 ng/kg) (Grover et al. 2011; Fuhrman et al. 2015) ranges). Using manure or slurry on agricultural fields is not only a traditional method, but also a part of the so-called circular economy. Due to this practice, however, steroidal estrogens (SE) can easily penetrate the soil and the groundwater (Zitnick et al. 2011), and can be taken up by plants and accumulated in their different parts (Erdal and Dumlupinar 2011; Gworek et al. 2021). Being already in the food chain, they can potentially cause adverse effects (e.g. fertility problems, feminising effects, cancer) on wildlife, cultivated livestock and human health alike (Grover et al. 2011; Fuhrman et al. 2015). The level of SE content can reach the hundreds, even up to the thousands of ng/kg magnitude (Zhang et al. 2015; Gudda et al. 2022). The estrogenic effect of 17ß-estradiol (17ß-E2) is 20,000 times higher than that of bisphenol-A (BPA), a widely known EDC of industrial origin (Coldham et al. 1997).

The main objective of this research was to study the environmental “price” of large-scale, continuous milk production from a rarely known perspective, i.e. mapping the estrogenic footprint (the amount of *oestrus-*inducer hormonal products, and that of the generated endoestrogens) in the slurry, produced at a dairy cow farm. To our knowledge, the detectability and the decomposition of *oestrus*-inducer hormonal products in slurry during four consecutive years have not yet been studied. The present study investigates the fate of 5 *oestrus*-inducing veterinary products used in a real dairy cow farm in Hungary. We examined the use of these OIVPs from 2017 to 2020 and tested their estrogenic activities as well.

For testing the estrogenic effects, we applied a dual approach: for testing exact molecules, an ultra-high-performance liquid chromatography (UHPLC-FLD) method was used. However, we performed a methodology development and validation to have a reliable effect-based method (EBM) for more holistic results and the Yeast Estrogen Screen (YES) test was our main research method (adapted from ISO 19040 standard). It employs the genetically modified *Saccharomyces cerevisiae* BJ3505 strain, which contains human estrogenic receptor. The YES test is largely able to overcome the difficulties caused by complex biological matrices and the chemical diversity of the molecules to be tested (Jobling et al. 2009).

A compound can be considered a material with estrogenic effect if it can bind to the estrogen receptors (ER) and is able to generate biological effects (Bittner et al. 2014). Numerous pollutants occur in the environment that can bind to those receptors (Arya et al. 2020). The simultaneous presence of antibiotics and estrogens means greater ecological risk than as if they were separate pollutants because antibiotics may increase the persistence of estrogens (He et al. 2019).

The appearance of SEs in the environment has attracted scientific interest worldwide. These studies mainly focused on SEs appearing in the aquatic environment or in wastewater treatment facilities (Arlos et al. 2016; Cerná et al. 2022) coming from urban areas or from animal husbandry (Du et al. 2020; Lin et al. 2020; Zhong et al. 2021). According to He et al. (2019), as much as 90% of ambiently appearing estrogens come from animal husbandry. The estimated total animal-borne estrogen emission of the European Union and the USA is 83,000 kg/*annum*, more than twice as much a human emission (Shrestha et al. 2012; Laurenson et al. 2014). Johnson et al. (2006) estimated that a dairy cow releases 384 mg 17ß-estradiol (17ß-E2) into the environment with *urine* and *faeces* on a daily basis, while a sow in farrow excretes 700–17,000 mg estron (E1) daily.

Besides the estrogens produced by the living organisms themselves (endoestrogens), there are several types of organic and inorganic molecules that are able to recognise the ligand bonding domains of the estrogen receptors (Farooq 2015). Endoestrogens are synthesised by the genitals and other organs from cholesterol, such as estrone (E1), estradiol (17ß and 17α E2) and estriol (E3) (Farooq 2015), while xenoestrogens are synthetic compounds that have estrogenic effects, e.g. pharmaceuticals.

## Materials and methods

### Description of the cattle farm

The farm studied is situated near Budapest, Hungary. The livestock consists of 2500 animals on average, out of which 1600 are active dairy cows, while the rest are dry cows, heifers and calves. There are 1500 calvings/*annum* on average. Manure is collected as slurry in a large storage pool. The average amount of slurry is 70,000 m^3^/*annum*. It is spread onto agricultural fields depending to the availability of the sites.

### Propagation protocol of the farm

Reproduction of cattle is under the regulation of many hormones. Reproduction is directly regulated by the Gonadotropin-releasing hormone (GnRH) which is secreted from the *hypothalamus*; estrogens secreted from the follicles, progesteron secreted from the *corpus luteum* and prostaglandin (F2α; PGF2) secreted from the *endometrium* in the inner part of the *uterus*. All the hormones listed have their own functions as well as influence on other hormones during the entire reproduction cycle of the cow (Sammad et al. 2019).

Managements often shift to targeted breeding programmes to simplify their lives. One of them is based on the application of PGF2α with which cows can be inseminated at any desired time, without the need for a subjective decision on the *oestrus*. For the synchronisation of the cycle and to influence the growth and the development of the *follicle*, hormonal (GnRH) products are used (Gábor et al. 2004; Ricci et al. 2020). The so-called OvSynch protocol was employed at the farm studied in 2017 and in the first half of 2018 as the base programme of the reproduction biology. In the second half of 2018, they introduced the double OvSynch protocol during which the animal goes through a second OvSynch protocol again with a 7-day delay and receives insemination only after that. In the latter case, everything is timely programmed, it simply makes the cow ready for insemination, but it needs precision (Yániz et al. 2004; Dirandeh et al. 2015; Nowicki et al. 2017).

Description of the *oestrus*-inducing veterinary pharmaceuticals.

Given that the lactation of dairy cows starts only after calving, successful insemination is inevitable for successful milk production throughout the year, distributed among the entire livestock. At large farms, it is programmed with intramuscularly applied hormonal injections. In our research, we examined the 5 different OIVPs (Table 1) that were actually used at the farm.

**Table 1:** Technical data of the 5 *oestrus*-inducing veterinary pharmaceuticals used at the farm

	Ovarelin	Gonavet	PGF	Alfaglandin	Dinolytic
Main ingrediens	0.050 mg/ml D-Phe6-gonadorelin (form ofdiacetate-tetrahydrate)	0.050 mg/ml D-Phe6-gonadorelin	0.0875 mg/ml chloprostenol (equivalent to 0.092 mg/ml chloprostenol-sodium)	0.250 mg/ml chloprostenol (in the form of chloprostenol-sodium)	5 mg/ml dinoprost (as dinoprost-trometamin)
Auxiliary ingrediens	15 mg/ml benzyl-alcohol (E1519)	1 mg/ml chlorocresol	1 mg/ml chlorocresol	1 mg/ml chlorocresol	16.5 mg/ml benzyl-alcohol
Producer	Ceva Sante Animale, France	Veyx-Pharma GmbH, Germany	Veyx-Pharma GmbH, Germany	Alfasan Nederland B.V., Netherlands	Zoetis Belgium SA, Belgium
Targeted species	Cattle (cow, heifer)	Cattle, swine, horse	Cattle, swine	Cattle	Cattle, swine, horse
Dosage	2 ml/animal	2 ml/ind	5.7 ml/ind	2 ml/ind	5 ml/ind
Withholding period	0 day	0 day	in the case of milk: 0 day in the case of edible tissues: 2 days	in the case of meat and other edible tissues: 1 day in the case of milk: 0 day	in the case of meat and edible issues: 1 day, in the case of milk: 0 day

Sources of the data: official descriptions in the packagings of the products

### Taking and preparation of the slurry samples

Between 2017 and 2020, we regularly sampled the large slurry pool of 14,000 cubic metres on a quarterly basis. Every time, samples were taken using 4 disposable, sterile, polypropylene centrifuge vials of 50 ml each, which were free from DNAase, RNase, endotoxins and metals, could be frozen to − 80 °C and were resistant to chemicals. Glassware involved in the test were washed thoroughly as usual in the laboratory practice, than were rinsed with ethanol twice and dried at 120 °C for 2 h. We took the subsamples from each corner of the slurry pool, mixed, then stored them at 4 °C in a refrigerator and processed them within 1–3 days after sampling. The sample containing vials were centrifuged for 20 min at 4 °C and at 4200 rpm (Heraus Megafuge 40 R centrifuge), during which the liquid and the solid phases of the slurry were separated. After that, materials with estrogenic effect were extracted from the supernatant using solid phase extraction (SPE), and from the solid phase, after a separation method.

SPE is one of the best options and therefore often used to extract and concentrate analytes of interest from complex biological matrices. For our research, OASIS HLB 6 cc 200 mg 30 µm cartridge was used. First, we conditioned the column with 8 ml pure methanol and with 8 ml water: methanol 95:5 mixture. Secondly, we loaded the sample by adding 30 ml supernatant to the cartridge. Thirdly, we washed the impurities from the column using 10 ml water:methanol 1:1 mixture and water:acetone 2:1 mixture. After drying it for 1 min, we eluted the analytes with 5 ml pure methanol. The eluent contained the materials with estrogenic effect and was ready for the yeast test. We measured 10 µl from it into each well of the 96-well plate for the YES tests and 3 ml aliquots were preserved for the UHPLC analyses. The latter ones were frozen immediately to − 20 °C and were kept at that temperature in refrigerator.

From the solid phase of the slurry, we measured 2 g into beakers of 50 ml and added 10 ml pure methanol to each of them. After an ultrasonic treatment for 30 min at 30 °C (JEKEN PS 40A 10L) and a centrifuge stage at 2000 rpm, at 4 °C for 10 min, the supernatant was ready for the yeast test; therefore, we measured 10 µl into each well of the 96-well plate. Three millilitre aliquots were preserved for the UHPLC analyses. The latter ones were frozen to − 20 °C and were kept at that temperature in refrigerator.

### Preparation of the OIVPs for the testing of estrogenic effect

We attempted to test the estrogenic effect of the pure OIVPs which were used at the dairy cow farm by performing the in vitro yeast assay on them. For this reason and to avoid contamination, an intact bottle from each of the 5 OIVPs was transported into the laboratory. From all 5 types, the following series of volumes were measured undiluted into the wells of the 96-well plate: 20 µl, 10 µl, 5 µl, 1 µl, 0.5 µl, 0.1 µl. Number of repetitions: 4.

### Development, validation and implementation of the yeast test

The yeast test was developed from ISO 19040–1:2018 standard designed for measuring the estrogenic potentials of water, wastewater and sediment samples using *Saccharomyces cerevisiae* BJ3505 genetically modified yeast strain. The method was adapted to test our medicine and slurry samples. The Yeast Estrogen Screen (YES) test is a reporter gene analysis which serves to measure the activation of the human estrogen receptor-alpha (hERα) in the presence of compounds that generate estrogenic effect. If the yeast meets estrogenic molecules or homologue ones, it starts to produce the ß-D-galactosidase enzyme. The amount of the enzyme can be quantified by adding a yellow substrate, chlorophenolred-ß-D-galactopyranoside (CPRG), and measuring the resulted product of red colour at 580 nm with a spectrophotometer (Labsystems Multiskan MS) (Hong 2012).

On day 1, we started to breed the yeast in a breeding solution. With permanent stirring, it was kept at 30 ± 1 °C for 22 ± 1 h (incubator type: PLO-EKO Aparatura).

On day 2, we prepared the above-mentioned medicine and slurry (supernatant and sediment) samples and measured them into the 96-well plate, in four repetitions in each case. After drying them out, 80 µl of 0.3% etanol solution and 40 µl of yeast suspension were measured into the wells (the row of blanks served as negative control, getting the same treatment but without the testing organism, the yeast). The row of dilutions was kept in the above-mentioned incubator in the same conditions.

On day 3, samples were resuspended with pipettes and cell density was measured at 620 nm with a spectrophotometer to check whether and how the yeast grew homogeneously. Then, 30 µl was measured from each sample into a new plate; 50 µl Lac-Z reagent was added to each which contained CPRG reagent. After 1 h of incubation, we measured the colour changes at 580 nm wavelength (Purvis et al. 1991; Routledge and Sumpter 1996).

From the cell density measured at 620 nm and the colour changes measured at 580 nm, using Microsoft Excel and MyAssays Desktop softwares, we calculated the relative growth of the yeast, the average corrected absorbance, the inductive quocient, the limit of quantitation (LOQ), the limit of detection (LOD) and the lowest ineffective dilution (LID). A dose–response curve was established for a reference compound, 17β-estradiol, and this curve served as a benchmark for estrogenic activity. Statistical techniques were used to fit a curve to the experimental data points and the sigmoidal (S-shaped) curve, modeled using the four-parameter logistic function, was used for this purpose (Findlay and Dillard 2007; Hong 2012). Once the curve was fitted, it was used to estimate the estrogenic activity of the sample at specific concentrations. This interpolation allowed to determine if the sample exhibits estrogenic activity and used to identify whether a compound or sample surpasses a predefined estrogenic activity threshold. If the sample’s activity crossed the threshold (reached the linear stage of the dose–response curve), it is considered to possess estrogenic properties. In cases where a sample exhibited high estrogenicity, samples were diluted to capture the linear stage of the dose–response curve, ensuring more accurate measurement. The resulted EEQ concentration shows that the estrogenic activity of the sample is equivalent to the estrogenic activity of a 17ß-E2 solution with the same concentration.

### Description of the UHPLC method

For implementing the UHPLC analyses, frozen samples preserved from the YES tests were transported to a professional analytical laboratory belonging to the Hungarian Academy of Sciences. The thawed samples were filtered through glass fiber membrane filter (Chromafil GF/PET-45/25 (0.45um)) to avoid suspended pollutions. 0.5–1.0 ml aliquots were inserted into the autosampler.

Concentrations of pharmaceuticals in the liquid phase were analysed via UHPLC (Shimadzu-Nexera X2 LC-30AD) using fluorescence (FLD) and PDA detectors. Sensitive analytical methods were developed for the simultaneous determination of pharmaceuticals and estradiols in prepared samples. The excitation and emission wavelengths were 280 nm and 310 nm, respectively. For separation, reverse phase column was used (Kinetex C18; particle size: 2.6 μm; length: 150 mm). The mobile phase was 57:43% mixture of ultrapure water (acidified with 10 mM H_3_PO_4_) and acetonitrile. The flow rate was between 0.6 and 0.8 ml/min at 40 °C. The injected sample volume was 1 μl. For the limit of detection (LOD) and the limit of quantitation (LOQ), see Table 2. The chemicals (acetonitrile and methanol) and the standards of analytical purity (E1, 17α-E2, 17ß-E2, EE2, E3, gonadorelin, cloprostenol, dinoprost-trometamin, chlorocresol, benzyl-alcohol) were purchased from Sigma-Aldrich. Ultrapure water with a quality of 0.055 μS/cm (LaboStar® PRO TWF) was used in all the analytical procedures. The stock solutions were diluted in methanol and prepared in amber-stained borosilicate beakers. Calibration was carried out by the use of pharmaceuticals as external standards. The following concentrations were used in triplicate for each pharmaceutical: 1, 10, 50, 100, 500, 700 and 1000, µg/l.

**Table 2:** UHPLC measurement parameters for estrogenic hormones and the major and auxiliary ingredients of the drugs used

	Flow rate (ml/min)	Capacity factor (k′)	UV λ (nm)	LOD (ng/l)	LOQ (ng/l)
E3	0.7	2.7	-	1.7	5
EE2	0.6	2.9	-	2.27	6.89
17ß-E2	0.7	2.6	-	1.4	4.3
17α-E2	0.7	2.8	-	1.8	6
E1	0.7	2.7	-	1.6	4.9
D-Phe6-gonadorelin	0.8	2.5	-	15.5	26.5
Cloprostenol	0.8	2.5	-	15.7	27.1
Dinoprost-trometamin	0.8	2.5	-	16.2	28.3
Chlorocresol	0.8	2.5	-	15.9	27.3
Benzyl-alcohol	0.8	2.5	-	15.6	26.4

*E1* estrone, *17α-E2* 17α-estradiol, *17ß-E2* 17ß-estradiol, *EE2* ethynilestradiol, *E3* estriol

Most endrocrine disruptors have aromatic moieties that allow them to be distinguished by fluorescent detection, especially in the case of estradiols (Fig. S1.). Therefore, methods were carefully designed to achieve a suitable resolution with the fluorescence detector for each compound (Fig.S1, Fig. S2.). Fluorescence detection–coupled HPLC is an ideal tool for the routine measurement of estrogens due to its sensitivity, selectivity, and cost-effectiveness.

### Methods of statistical analyses of the results

We attempted to find correlations between two response variables (the estrogenic effect of the liquid and the solid phases of the slurry, averaged quarterly in µg/kg) and three groups of the background variables summed quarterly: (1) amount of injected active ingredients (mg) per active ingredient (D-Phe6-gonadorelin, chloprostenol, dinoprost-trometamin) and summed; (2) number of treatments per medicine (Ovarelin, PGF, Gonavet, Dinolytic, Alfaglandin) and summed; (3) auxiliary background variables (number of inseminations, number of calvings, number of dead calvings, number of abortions).

During the analysis of the row of data (*n* = 16), first, we quantified the correlation relationship among all the variables with the Pearson correlation coefficient. Then, we performed principal component analysis (PCA). During the PCA, we used the two response variables describing the estrogenic effect to stretch out the ordination space, and we fitted the background variables describing the active ingredients in that space with a permutation method using 1000 repetitions. Analyses were performed with “R” statistical software (R CORE TEAM 2020) and its “vegan” package (Oksanen et al. 2020).

## Results

### Validation of the UHPLC method

Linearity, precision (repeatability), accuracy (recovery) and limits of detection (LOD) and quantification (LOQ) were evaluated to verify the performance of the method. Validation was performed according to International Conference on Harmonisation (ICH) guideline Q2 (R1). The linearity was determined from 10 analytical curves in triplicates of the standards in the range of 0.001–2 µg/ml. The results were evaluated using the regression coefficients (Table S1, Table S2). To assess the precision and accuracy of the method, three concentrations (0.01–1 µg/mL) were measured at three different times of a same day and on three consecutive days. The results were expressed with relative standard deviation (RSD). The developed chromatographic method should provide separation of pharmaceuticals in the liquid phase of slurry. Peak interference was not observed during the analysis. Samples were spiked with the appropriate chemicals, especially when dissolved organic materials made any noise on the chromatogram. The analysis performed with the same equipment by a single analyst. The limits of detection (LOD) and quantification (LOQ) were determined based on the calibration curves. The standard deviation of y-intercepts of regression line was used as the standard deviation.

### UHPLC tests

Using a UHPLC-FLD method, we tested 32 slurry samples for 5 steroidal estrogen compounds, 3 active substances of *oestrus*-inductive pharmaceuticals and 2 auxiliary ingredients. At least one compound belonging to the estrogenic group could be detected from every sample. Out of the active substances of the OIVPs (D-Phe6-gonadorelin, cloprostenol and dinoprost-trometamin) and the two auxiliary ingredients studied (chlorocresol, benzyl-alcohol), all were under the detection limit in all samples. E1 and EE2 were also under LOD. By contrast, E3 was detected in 100% of the samples, 17α-E2 was in 78%, while 17β-E2 was in 66% of them. 17β-E2 and 17α-E2 appeared regularly at above-LOD levels in the samples from the second half of 2018. Values were steadily higher in the solid fraction of the slurry: in the case of 17β-E2, concentrations were six to seven times higher than in the liquid fraction (Table 3).

**Table 3:** The estradiol-equivalents (EEQ) values of hormonal drugs (from the YES test)

	2017				2018				2019				2020			
	Liquid (µg/l)	*SD*	Solid (µg/kg)	*SD*	Liquid (µg/l)	*SD*	Solid (µg/kg)	*SD*	Liquid (µg/l)	*SD*	Solid (µg/kg)	*SD*	Liquid (µg/l)	*SD*	Solid (µg/kg)	*SD*
1st	139.2	± 15	1337.0	± 36.5	123.9	± 9.3	1355.0	± 37.5	137.2	± 12.3	2745.0	± 240	248.1	± 15.7	2730.0	± 195
2nd	61.7	± 1.6	2555.5	± 190.5	88.9	± 2	1900.0	± 37.5	156.5	± 13.3	1990.0	± 19.5	196.5	± 9.7	3490.0	± 205
3th	19.3	± 1.1	1415.0	± 135.5	225.8	± 19.9	4570.0	± 250.5	193.1	± 9.8	3200.0	± 42.5	288.0	± 20.3	6300.0	± 245
4th	19.6	± 1.4	142.0	± 8	213.8	± 17	3970.0	± 255	271.1	± 15	4990.0	± 85	373.0	± 22.3	5325.0	± 285
Average per year	***60.0***		***1362.4***		***163.1***		***2948.8***		***189.5***		***3231.3***		***276.4***		***4461.3***	

### Validation of the yeast assay (YES test)

For the yeast test, LOQ was 1 ng/l 17ß-E2, while the LOD was 27 pg/l. To assess the accuracy and the precision of the tests, we tested 17ß-E2 standards in the concentrations ranging from 2 to 500 ng/l on 96-well plates. The precision was above 84%, which suits the Industrial Guideline for the Validation of Bioanalytical Methods. Accuracy, on the other hand, did not exceed the level of 80% for the two lowermost dilutions; therefore, only concentrations between 18.6 and 500 ng/l meet the requirements (for 2 ng/l: 48.8%; 6.2 ng/l: 76%; 18.6 ng/l: 91.3%; 55.6 ng/l: 91%; 166.5 ng/l: 97.9%; 500 ng/l: 98.3%, at 95% confidence).

### Slurry estrogenic effect test (YES test)

We revealed that the EEQ values of the slurry had a steadily rising tendency over time, with some seasonal fluctuations (Table 3). The compounds with estrogenic effects tend to bind more strongly to the solid phase than to the liquid part. The shift in the protocol at the farm in the 2nd quarter of 2018 doubled the number of hormonal injections administered. As a consequence, EEQ values referring to the liquid phase below 100 µg/l can only be found before that date (e.g. 4^th^ quarter of 2017); later on, only 2–3 times higher values can be observed. The solid phase, on the other hand, showed significantly higher EEQ values, greater by orders of magnitude than those of unpolluted samples. Values equal to or below 2000 µg/l can only be detected before the date of the protocol change. Later on, except for one piece of data, we measured up to 2–3 times higher EEQ values. Values measured in the 3rd and 4th quarters of the years of the research are typically higher than those in the 1st and 2nd quarters.

### Medicine active ingredient test (YES test)

We studied 5 *oestrus*-inducer hormonal products (Ovarelin, PGF, Gonavet, Dinolytic, Alfaglandin) by measuring 6 different volumes (20 µl, 10 µl, 5 µl, 1 µl, 0.5 µl, 0.1 µl) from each. We considered the estrogenic effect as the average of the responses gained for the six measurements. The lowest volume (0.1 µl) of either of the OIVPs did not show results during the yeast test. There are three active substances (D-Phe6-gonadorelin, chloprostenol, dinoprost-trometamin) in the 5 OIVPs, formulated with either of the two auxiliary ingredients (originally functioned as preservatives, i.e. benzyl-alcohol and chlorocresol). It is observable from Table 4 that even though neither of these hormones belong to the group of endoestrogens, all 5 showed estrogenic effect in the yeast test. It indicates that owing to their chemical structure, they are able to bind to the human estrogen receptor (hERα).

**Table 4:** EEQ values of hormonal drugs at indicated volumes. Normalised EEQ values refer to 1 mg medicine

Examined volume	Ovarelin	SD	PGF	SD	Gonavet	SD	Dinolytic	SD	Alfaglandin	SD
EEQ 20 µl	103.5	± 1.5	1099.5	± 4.7	469.5	± 3.1	127,5	± 1.7	1225.5	± 4.5
EEQ 10 µl	100.6	± 0.5	606	± 3.7	456	± 4.3	123	± 1.1	603	± 1.5
EEQ 5 µl	87.4	± 0.4	576.8	± 3.4	446.4	± 0.8	127.9	± 0.8	603.6	± 2.1
EEQ 1 µl	< LOD	-	1047	± 0.8	783	± 0.5	< LOD	-	516	± 0.6
EEQ 0.5 µl	< LOD	-	< LOD	-	< LOD	-	< LOD	-	732	± 0.6
Normalised EEQ value	9.7		83.2		53.9		12.6		73.6	

The veterinary medicine Alfaglandin contained 0.250 mg/ml chloprostenol as an active ingredient, while PGF only contained 0.092 mg/ml. We pointed out, however, that PGF showed higher estrogenic effect than Alfaglandin (83.23 µg/l vs. 73.6 µg/l, respectively). At a volume of 0.5 µl (0.5 µl/120 µl dilution in the 96-well plate), only Alfaglandin showed positive results, indicating that it keeps its estrogenic effect even at higher dilutions (from the point of view of environmental pollution, persistency is a negative characteristic). The estrogenic effect of D-Phe6-gonadorelin: The estrogenic effect of Gonavet was 5 times higher than that of Ovarelin (53.87 µg/l vs. 9.72 µg/l, respectively), even though both OIVPs contained the same D-Phe6-gonadorelin as an active ingredient and in the same concentrations (0.050 mg/ml). They only differed in the auxiliary ingredients: chlorocresol (1 mg/ml) and benzyl-alcohol (15 mg/ml), respectively. For testing the estrogenic effect of dinoprost-trometamin, only one veterinary product was available at the farm, Dinolytic, which contained 5 mg/ml active ingredient and showed 12.61 µg/l estrogenic effect. The results are similar to those of Ovarelin, which had the same auxiliary ingredient, a relatively high concentration of benzyl-alcohol (16.5 mg/ml).

### Analysis of reproduction biology

**Fig. 1:**
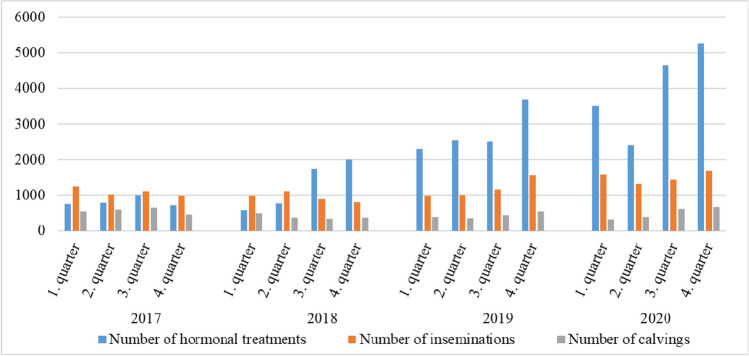
Reproductive indicators—numbers of hormonal treatments, inseminations and calvings. These factors have important functions in animal reproduction throughout the year

We evaluated the most important parameters of the reproduction biology of the farm during this research period which show the performance of the farm from a managerial perspective. The number of hormonal treatments was rising steadily at the farm from 2017, as it can be seen from the numbers of inseminations and calvings (Fig. 1). In the second half of 2018, there was a shift in the insemination protocol (OvSynch to Double OvSynch); therefore, the number of hormonal treatments doubled.

To provide a stable milk supply throughout the year and more independence from natural factors influencing milk production (and to generate more profit), the OIVPs studied were used in large amounts at the farm. The average number of treatments per dairy cow was 0.68 in 2017, 1.02 in 2018, 1.73 in 2019 and 2.2 in 2020. We revealed that not only did the number of hormonal treatments rise continuously from 2017, but also the estrogenic content (EEQ value) of the slurry. The rise of EEQ value in the resulting slurry between the starting (2017) and the closing year (2020) of our research was almost fourfold.

### Statistical analyses

**Fig. 2:**
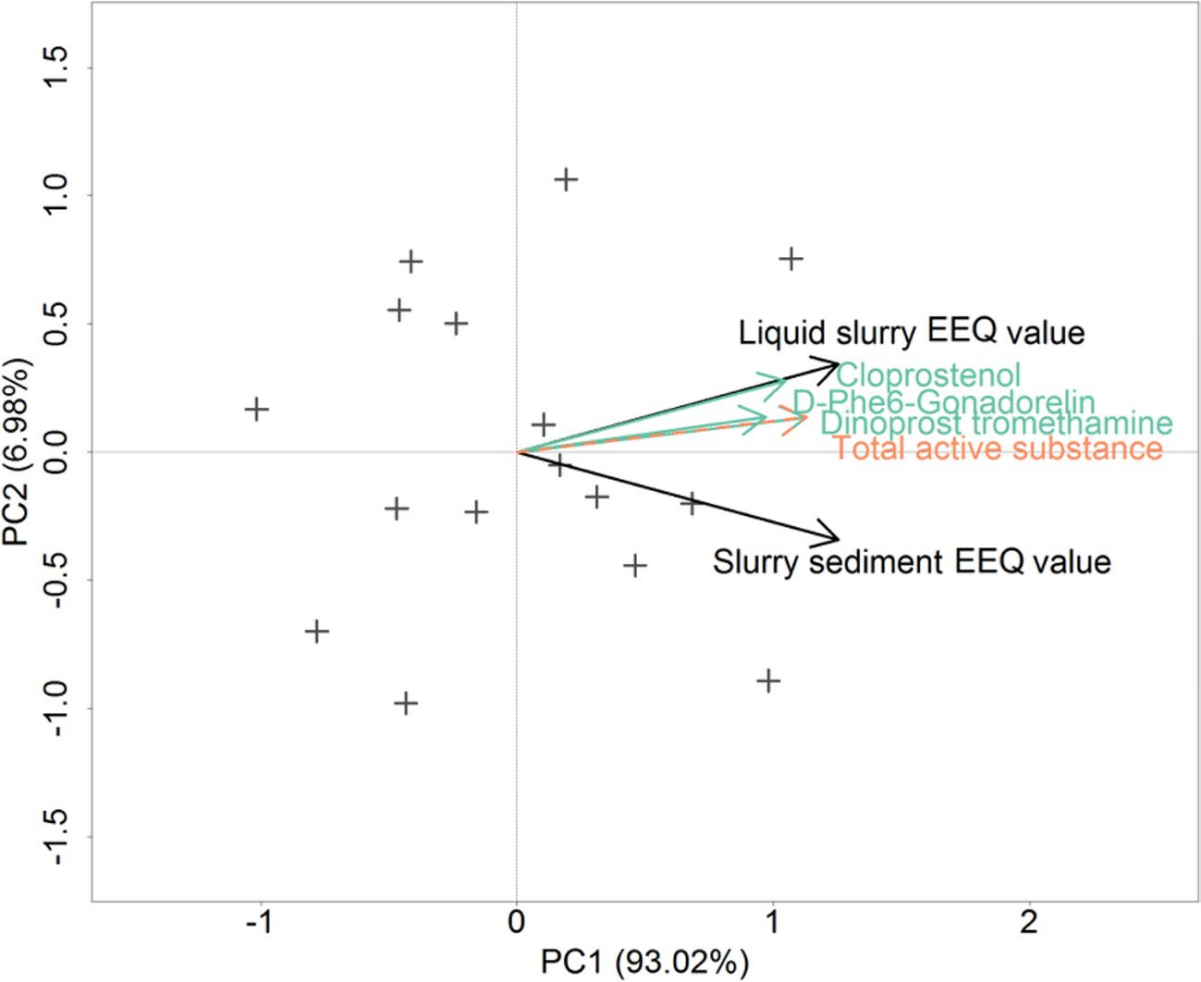
Principal component analysis (PCA): The ordination space created by the two response variables (liquid and sediment parts of slurry) and the background variables (active substances) are visible. + indicates the individual measurement times, the direction of the arrows shows with which principal component the given variable correlates the most and the lengths of the arrows show the magnitude of the correlation (We have marked the liquid and sediment fractions of the slurry in black, the active ingredients in green and the total active ingredients in orange)

The two response variables, namely the quarterly averaged estrogenic effects of the liquid and the solid parts of the slurry, are strongly correlated (*r* = 0.86, *n* = 16, *p* < 0.001). The estrogenic effect of the liquid phase shows a stronger correlation with the background variables (i.e. with the active substances, with the formulated OIVPs and with the auxiliary background variables) than that of the solid phase (Fig. 2).

According to the Pearson correlation, the estrogenic effect of the liquid phase has a strong positive and significant correlation (*p* < 0.001) with dinoprost (*r* = 0.86), chloprostenol (*r* = 0.83) and gonadorelin (*r* = 0.75). Their relationship with the estrogenic effect of the solid phase is slightly weaker and less significant (*p* < 0.01); their correlation coefficients are 0.81, 0.72 and 0.69, respectively.

The quarterly summed number of medical treatments shows a strong correlation with the estrogenic effect of the liquid (*r* = 0.89, *p* < 0.001) and the solid phases (*r* = 0.81, *p* < 0.001). Though slightly weaker, there is still a strong correlation between the estrogenic effect of the liquid phase and Dinolytic (*r* = 0.86, *p* < 0.001), Alfaglandin (*r* = 0.79, *p* < 0.001) and Gonavet (*r* = 0.75, *p* < 0.001), while PGF is non-significant and neutral (*r* = 0.08, *p* = 0.76) and Ovarelin shows a negative correlation (*r* =  − 0.61, *p* < 0.05). The correlations of the medical treatments with the estrogenic effect of the solid phase can be listed in the same order: Dinolytic: *r* = 0.81, *p* < 0.001; Alfaglandin: *r* = 0.68, *p* < 0.01; Gonavet: *r* = 0.67, *p* < 0.01; PGF: *r* = 0.10, *p* < 0.70; and Ovarelin: *r* =  − 0.55, *p* < 0.05.

From the auxiliary background variables, insemination showed slightly positive (*r* = 0.64), dead calving and abortion showed slightly negative (*r* =  − 0.43 and *r* =  − 0.37, respectively), while calving showed neutral (*r* = 0.04) correlation with the estrogenic effect of the liquid phase of the slurry, out of which only the correlation of the insemination is significant at 0.005 level of significance. The correlation of the auxiliary variables with the solid phase gives similar results; the only correlation that we found with marginal significance (*p* = 0.05) was in the case of insemination (*r* = 0.47).

Figure 2 shows the results of the PCA for the active substances. The ordination could comprise the major part, 93% of the variance into one principal component. The two response variables, i.e. the estrogenic effects of the liquid and the solid phases of the slurry, correlated with each other strongly, and just slightly turned away from this principle component. All active substances point to the direction of the estrogenic effect of the liquid phase. Chloprostenol most correlated to the liquid phase out all of them. The least specific seemed to be dinoprost and the total amount of active substances.

**Fig. 3:**
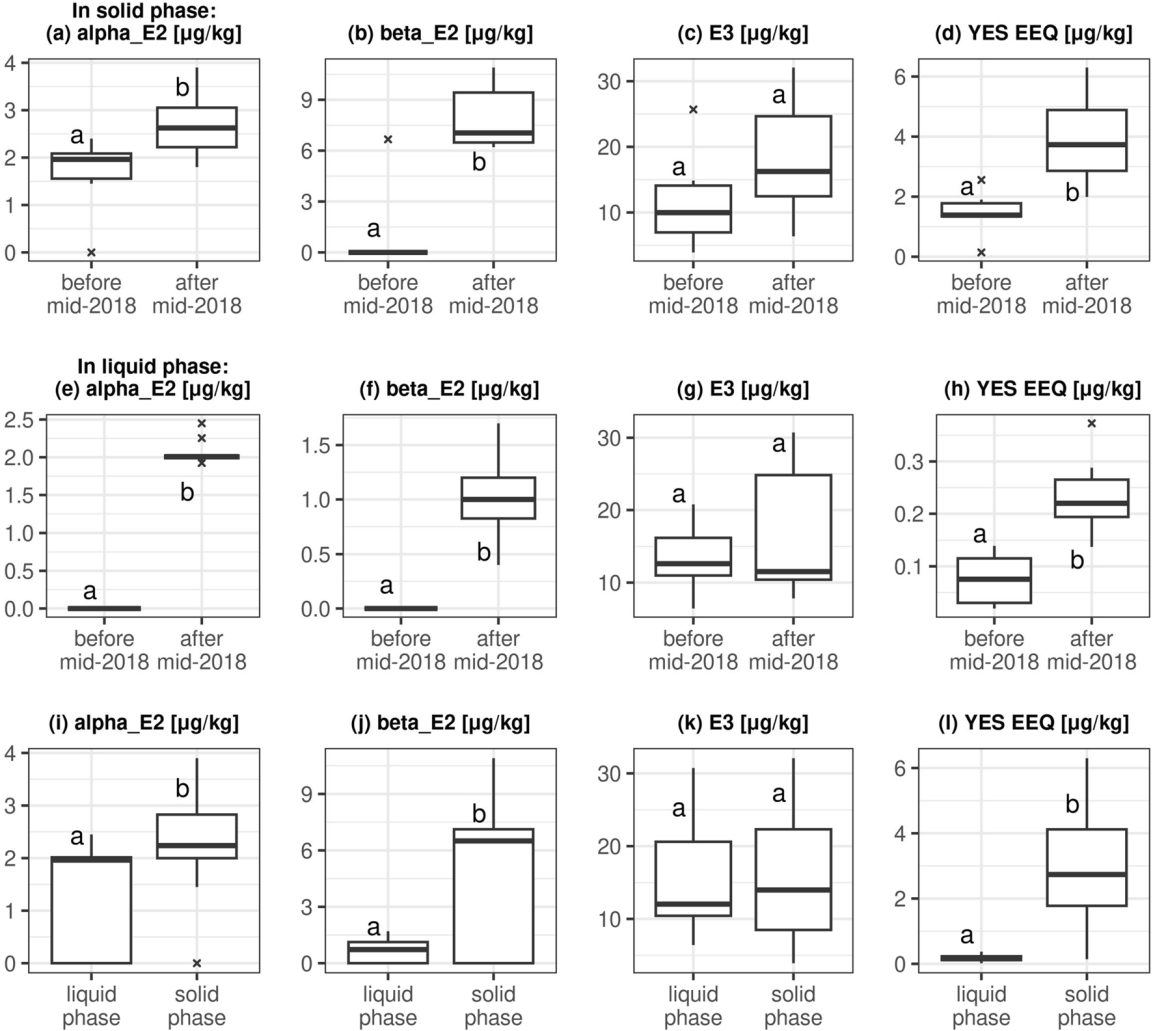
The results of the UHPLC and YES tests are shown. For each hormone investigated, statistical properties of the concentration and equivalent concentrations (EEQ) are shown. In the box plots, first and third quartiles and the median are represented as the lower and upper side of the box and the bold horizontal line, respectively. The vertical line extensions show minimum and maximum values at most 1.5 × IQR (interquartile range) from the box, where IQR is the difference between the third and the first quartiles. All values outside this range are considered outliers and are shown with bold x. The first and second row of plots show only results in the solid and liquid phases, additionally separating before and after the 2018 values. The third row of boxes shows all results separating solid and liquid phases per box plot. Results of a two-sample *t*-test with *p* = 0.05 are marked with mismatching (“a” and “b”) or matching (“a” and “a”) letters marking if a significant difference was found or not, respectively

We have also composed a complex figure comprising the chemical analyses of the results of several tests in box plots (Fig. 3). Horizontally: the upper row of boxes (a, b, c, d) represents results based on the solid phase of the slurry; boxes a, b and c show UHPLC and d shows the YES test results. The second row of boxes (e, f, g, h) comes from testing the liquid phase, while the lower row of boxes (i, j, k, l) shows the comparisons of the data measured in all the liquid and solid phase samples. Vertically (columns of boxes): boxes a, e and i, show UHPLC results for 17α-E2, boxes b, f and j show UHPLC results for 17ß-E2, while boxes c, g and k show UHPLC results for E3. The fourth column (d, h, l) shows the YES test results for the solid and the liquid phases, as well as the comparisons between the data measured before and after the protocol change. Tendencies clearly show that the protocol change raised the estrogenic content of the slurry and estrogenic compounds tend to bind more strongly to the solid phase.

## Discussion

The present field-based, longitudinal study investigates the fate of 5 different *oestrus*-inducer drugs from the use phase to their appearance in the slurry at a dairy cow farm in Europe, Hungary. We examined the use of these OIVPs from 2017 to 2020 and determined the estrogenic effects of the generated higher hormonal excretion of the cows appearing in the slurry as well.

It is a biological destiny that milk production starts only after calving. Therefore, manipulation of the reproduction biology of the cows targets the maximisation of the yearly milk production per dairy cow, distributed among the livestock throughout the year.

Our research just partially supports what Jobling et al. (2009) claimed in that results of the chemical analyses and those of the yeast tests are not comparable and estrogenic activities (EEQ values) of the samples do not correlate well with the measured analytical concentrations of the individual steroidal estrogens. Our results are more consistent: Using UHPLC analyses, we gained exact concentrations of *some* chemicals, while the YES test provides holistic data on the estrogenic effect of the whole sample, for *a number of* molecules. On Fig. 3, tendencies are the following: the protocol change in mid-2018 raised the concentrations of 17α-E2 and 17ß-E2 alike, both in the solid and liquid phases of the slurry, and the differences are statistically significant. E3 values were larger by one order of magnitude throughout the research period, but the visible rise is not significant. Given that E1 and EE2 results were under LOD, they are not represented on this figure. The YES test provided similar rising tendencies with significant difference in each case.

From an environmental perspective, however, we agree with Jobling et al. (2009): results of the yeast test can be considered more relevant because the hormone-like effects of all compounds as well as the interactions among the chemicals are observable. Therefore, these results have higher predictive values when it comes to measuring potentially important effects on human health (Jobling et al. 2009). That is the main reason why the yeast test was chosen for our research.

Even though some hormonal products contained the same *active ingredients*, they generated different estrogenic effects depending on their *auxiliary ingredients* (benzyl-alcohol vs. chlorocresol). The higher EEQ value we measured for a drug, the stronger hormonal effect it had. It is important to note that we gained these values with the co-presence of the active ingredient having a *known* hormonal effect and the auxiliary ingredient which was theoretically non-hormonal and served as preservative only. The co-presence of the two materials makes chemical/biochemical interactions (synergism or antagonism) between them possible.

It can be hypothesised from the data presented in Tables 1 and 3 that if a medicine with an equal or even lower hormonal level contains a synergistic auxiliary material (e.g. chlorocresol seems to be synergistic), it is able to generate the same or an even higher physiological effect (ovulation, successful insemination, higher milk production) than another drug which is even high in hormonal content but contains an auxiliary material (e.g. benzyl-alcohol) with a low or no synergistic effect. Once administered to the animal, the medicine works well, keeping its high hormonal effect, *imitating* high hormonal content, but the main and the auxiliary materials are metabolised in different ways, the synergistic effect ceases, and the medicine ends up as a low hormonal pollution in the manure or slurry. The pairing of Gonavet and Ovarelin suits our hypothesis: both have the same active substance at the same concentration, but their auxiliary materials differ (chlorocresol vs. benzyl-alcohol, respectively). We measured 5.54 times higher EEQ value for Gonavet, most likely because the auxiliary substance chlorocresol synergistically strengthened the estrogenic effect of the main ingredient. Our hypothesis is also supported by the fact that Alfaglandin kept its high hormonal effect even at high dilutions. In fact, at that level, only Alfagladin showed a positive estrogenic effect, due to its inherently high hormonal power plus the effect of the synergistic auxiliary ingredient. (At that dilution level, its hormonal level should have been ceased and be under LOD.) Even though only Dinolytic represented drugs with dinoprost as an active substance, indirectly it also supports our hypothesis, given that its EEQ value was low, indicating that its auxiliary ingredient, despite its very high concentration, did not perform a synergistic effect with the active substance.

The lesson learned from the above-mentioned examples, which can also be transformed into a strategic suggestion for the practice, is that it is advisable to choose a medicine which contains a synergistic auxiliary ingredient (chlorocresol) independent from the active substance. This step would reduce the ecological footprint and the risk of food contamination and human health problems of the artificial induction of the *oestrus* of dairy cows. In the light of our results, it is predictable that slurry can be less hazardous to the environment later on when it is applied on the field.

Limitations of the study: while the Yeast Estrogen Screen (YES) assay measures the activity of the human estrogen receptor, it is important to acknowledge that it cannot serve as a direct model for human cellular responses. This limitation arises from the inherent dissimilarities between yeast and human cells, primarily attributed to the presence of a protective cell wall in yeast. This cell wall significantly alters the manner in which external substances interact with and penetrate yeast cells, resulting in distinct cellular responses compared to human cells. In spite of its limitations, however, in the linear range of the calibration curve it does work well as a holistic method. In spite of its limitations, researchers appreciate EBM’s sensitivity (Itzel et al. 2019; Simon et al. 2022), cost-effectiveness and excellent screening capability in ecotoxicologically comprehensive water quality assessment, worth implementing EU-wide (Simon et al. 2022). Therefore, the need emerged among experts to incorporate EBMs (incl. the YES test) into the European Water Framework Directive.

Toxicological assessments are often restricted to immediate effects, e.g. oral acute toxicity, in a usual range of concentration (%, g/l, mg/l, etc.). Micropollutants, on the other hand, are effective at very low (ng/l) concentrations at which conventional instrumental analytical methods are not sensitive enough: LOQ values are often greater than the effective concentrations, meaning: by the time GC- and HPLC-based methods are able to provide reliable results, the chemicals studied are already able to cause adverse effect to humans or the environment.

By contrast, the YES test provides “exquisite sensitivity” to estrogens (Coldham et al. 1997). We believe that YES test can be a sensitive indicator of estrogenic activity of the environment to provide a warning signal ahead of time to avoid adverse effects on human health.

The studied drugs showed estrogenic effects according to the xenoestrogenic effect mechanism given that their structural *formuli* have some similarities to those of the compounds in the estrogenic group. Some ligands of the compounds used are able to bind to those platforms of the receptors which would make bonds with the adequate groups of the real estrogens. If we compare the binding of estrogens to their receptors to the traditional “key-lock” theory, we can realise that this case is a “fake key-lock” situation: chemically different compounds with similarities in their structures generate similar biological effects. In fact, when we measure the EEQ values of these materials, we gain a holistic result of the described biochemical process.

The Green Chemistry concept prefers the most benign and natural materials and methods available in order to prevent the formation of harmful products and wastes and to eliminate existing ones. Natural decomposing of EDCs, especially in an aquatic environment, is connected to aerobic conditions, sunlight (Kim et al. 2017), the presence of Fe (III) ions and dissolved organic matter (DOM) (Gu et al. 2019). The process can be artificially accelerated by activated carbon (Rovani et al. 2014), or by advanced oxidation processes (AOPs) such as with ozonation and H_2_O_2_ treatment (Esplugas et al. 2007; Wolf et al. 2022). However, considering their costs, they are only worthwhile in cases of drinking and wastewater treatment. Our research proved that there is a significant difference between the EEQ value (EDC content) of the liquid and the solid phases of the slurry and SEs tend to bind more to the solid phase (this finding is similar to that of Zitnick et al. (2011) who claimed that 17ß-E2 tends to bind strongly to soils and sediments). It means that in the practice, the use of separators is feasible, and the resulting liquid phase can be applied as irrigation water fairly safely, while treating the solid phase with natural processes (exposure to sunlight and atmospheric oxygen, application of some benign additives) seems more suitable and economically viable, while still being conscious about the possible release of the valuable nitrogen content. Papers reporting studies on manure usually focus on nutrient content and utilisation only, ignoring the possibility of hormonal pollution of agricultural fields with the EDC content. Detailed studies of the elimination processes, involving the expensive 14C-labelled SE molecules (Ian et al. 2019), or the study of uptaking of EDCs by plants on the sites is an interesting topic for further research but is beyond the scope of the present paper.

## Conclusions

The first principle of Green Chemistry warns us that preventing the formation of wastes is much better, cheaper and more sustainable than treating or cleaning an already polluted material or a site in the environment. Our study reveals that intensifying breeding practices in dairy cow farms generate the hidden risk of hormonal pollution in agricultural fields or in the environment. This, through the food chain, may cause adverse effects to the wildlife and humans as well, which can appear as reduced reproductive fitness. That can be behind the infertility problems of the growing number of couples worldwide.

Due to the potentially large number of hormonal metabolites, UHPLC-based methods alone cannot describe the potential risk of samples, but the modified YES test we worked out provide a feasible solution for testing, anywhere in the world.

We concluded that the simplest way to reduce the hormonal effect of the slurry is choosing the right medicine, in which main and auxiliary ingredients are combined to utilise synergistic effect in reducing the level of the hormonal pollution. Meanwhile, we suggest further research on OIVPs by experts of veterinary medicines.

Our research underlines that slurry is a kind of material which, before applying on the field, should be treated with new methods, such as separation and composting, due to its hormonal content, not only because of environmental pollution but also because of human health risks.

Our research proves that the ecological footprint of artificial hormonal treatments can be reduced by raising not the real but the visible or imitated hormonal effect of the injection. To find a robust, cheap and reliable testing method, the YES test, as our research establishes, proves a good option. Beyond its feasibility, its environmental footprint is very small, which is a great advantage from the point of view of the Green Chemistry concept.

### Supplementary Information

Below is the link to the electronic supplementary material.

Supplementary file1 (DOCX 150 KB)

## Data Availability

The raw experimental data are available from EG upon request.

## References

[CR1] Arlos MJ, Liang R, Hatat-Fraile MM, Bragg LM, Zhou NY, Servos MR, Andrews SA (2016). Photocatalytic decomposition of selected estrogens and their estrogenic activity by UV-LED irradiated TiO_2_ immobilized on porous titanium sheets via thermal-chemical oxidation. J Hazard Mater.

[CR2] Arya S, Dwivedi AK, Alvarado L, Kupesic-Plavsic S (2020). Exposure of U.S. population to endocrine disruptive chemicals (Parabens, Benzophenone-3, Bisphenol-A and Triclosan) and their associations with female infertility. Environ Pollut.

[CR3] Bittner GD, Denison MS, Yang ChZ, Stoner MA, He G (2014). Chemicals having estrogenic activity can be released from some bisphenol a-free, hard and clear, thermoplastic resins. Environ Health.

[CR4] Cerná T, Ezechiás M, Semerád J, Grasserová A, Cajthaml T (2022). Evaluation of estrogenic and antiestrogenic activity in sludge and explanation of individual compound contributions. J Hazard Mater.

[CR5] Coldham NG, Dave M, Sivapathasundaram S, McDonnell DP, Connor C, Sauer MJ (1997). Evaluation of a recombinant yeast cell estrogen screening assay. Environ Health Perspect.

[CR6] Dirandeh E, Roodbari AR, Colazo MG (2015). Double-Ovsynch, compared with presynch with or without GnRH, improves fertility in heat-stressed lactating dairy cows. Theriogenology.

[CR7] Du B, Fan G, Yu W, Yang S, Zhou J, Luo J (2020). Occurrence and risk assessment of steroid estrogens in environmental water samples: a five-year worldwide perspective. Environ Pollut.

[CR8] Erdal S, Dumlupinar R (2011). Mammalian sex hormones stimulate antioxidant system and enhance growth of chickpea plants. Acta Physiol Plant.

[CR9] Esplugas S, Bila M, Kraues LGT, Dezotti M (2007). Ozonation and advanced oxidation technologies to remove endocrine disrupting chemicals (EDCs) and pharmaceuticals and personal care products (PPCPs) in water effluents. J Hazard Mater.

[CR10] Farooq A (2015). Structural and functional diversity of estrogen receptor ligands. Curr TopMed Chem.

[CR11] Findlay JWA, Dillard RF (2007). Appropriate calibration curve fitting in ligand binding assays. AAPS J.

[CR12] Fuhrman VF, Tal A, Arnon S (2015). Why endocrine disrupting chemicals (EDCs) challenge traditional risk assessment and how to respond. J Hazard Mater.

[CR13] Gábor GY, Tóth F, Szász F, Petró T, Györkös I (2004). Possibilities of the shortening of the timeslot between two calvings in dairy cow stocks. Oestrus inductive and ovulation syncronising methods. J Hungarian Vet.

[CR14] Grover DP, Zhou JL, Frickers PE, Readman JW (2011). Improved removal of estrogenic and pharmaceutical compounds in sewage effluent by full scale granular activated carbon: impact on receiving river water. J Hazard Mater.

[CR15] Gu L, Huang B, Han F, Xu Z, Ren D, He H, Pan X, Dionysios DD (2019). Intermittent light and microbial action of mixed endogenous source DOM affects degradation of 17β-estradiol day after day in a relatively deep natural anaerobic aqueous environment. J Hazard Mater.

[CR16] Gudda FO, Ateia M, Waigi MG, Wang J, Gao Y (2022). Ecological and human health risks of manure-borne steroid estrogens: a 20-year global synthesis study. J Environ Manag.

[CR17] Gworek B, Kijenska M, Wrzosek J, Graniewka M (2021) Pharmaceuticals in the soil and plant environment: a review. Water, Air, and Soil Pollution 232(4). 10.1007/s11270-020-04954-8

[CR18] He Y, Wang T, Sun F, Wang L, Ji R (2019). Effects of veterinary antibiotics on the fate and persistence of 17β-estradiol in swine manure. J Hazard Mater.

[CR19] Hong LTA (2012) The YES assay as a tool to analyse endocrine disruptors in different matrices in Vietnam. Institut für Nutzpflanzenwissenschaften und Ressourcenschutz (INRES) Lehr-und Forschungsbereich Pflanzenernährung der Rheinischen Friedrich-Wilhelms-Universität zu Bonn. https://d-nb.info/1043056491/34

[CR20] Ian X, Wang T, Ewald F, Chen Z, Cui K, Schaffer A, Wang L, Ji R (2019). 14C-labelling of the natural steroid estrogens 17α-estradiol, 17β-estradiol, and estrone. J Hazard Mater.

[CR21] ISO 19040–1:2018 Water Quality (2018) Determination of the estrogenic potential of water and waste water - part 1: Yeast Estrogen Screen, Saccharomyces Cerevisiae.

[CR22] Itzel F, Gehrmann L, Teutenberg T, Schmidt TC, Tuerk J (2019). Recent developments and concepts of effect-based methods for the detection of endocrine activity and the importance of antagonistic effects. Trends in Analytical Chem.

[CR23] Jobling S, Burn RW, Thorpe K, Williams R, Tyler C (2009). Statistical modeling suggests that antiandrogens in effluents from wastewater treatment works contribute to widespread sexual disruption in fish living in English rivers. Environ Health Perspect.

[CR24] Johnson AC, Williams RJ, Matthiessen P (2006). The potential steroid hormone contribution of farms animals to freshwaters the united kingdom as a case study. Sci Total Environ.

[CR25] Kim S, Cho H, Joo H, Her N, Han J, Yi K, Kim JO, Yoon J (2017). Evaluation of performance with small and scale-up rotating and flat reactors; photocatalytic degradation of bisphenol A, 17–estradiol, and 17–ethynyl estradiol under solar irradiation. J Hazard Mater.

[CR26] Laurenson JP, Bloom RA, Page S, Sadrieh N (2014). Ethinyl estradiol and other human pharmaceutical estrogens in the aquatic environment: a review of recent risk assessment data. AAPS J.

[CR27] Li C, Li Y, Li X, Ma X, Ru S, Qiu T, Lu A (2020) Veterinary antibiotics and estrogen hormones in manures from concentrated animal feedlots and their potential ecological risks. Environ Rese 110463. 10.1016/j.envres.2020.110463 10.1016/j.envres.2020.110463 33189740

[CR28] Lin CY, Gong J, Zhou YS, Chen DY, Chen YH, Yang J, Li Q, Wu CQ, Tang HM (2020). Spatiotemporal distribution, source apportionment, and ecological risk of corticosteroids in the urbanized river system of Guangzhou, China. Sci Total Environ.

[CR29] Nowicki A, Baranski W, Baryczka A, Janowski T (2017) OvSynch protocol and its modifications in the reproduction management of dairy cattle herds – an update. J Vet Res 61(3)*.*10.1515/jvetres-2017-0043 10.1515/jvetres-2017-0043 PMC5894419 29978091

[CR30] Oksanen J, Blanchet FG, Friendly M, Kindt R, Legendre P, McGlinn D, Minchin PR, O’hara RB, Simpson GL, Solymos P, Stevens MHM, Szoecs E, Wagner H (2020) Vegan: community ecology package. R package version 2:5–7. https://CRAN.R-project.org/package=vegan

[CR31] Purvis IJ, Chotai D, Dykes CW, Lubahn DB, French FS, Wilson EM, Hobden AN (1991). An androgen-inducible expression system for Saccharomyces cerevisiae. Gene.

[CR32] R Core Team (2020) R: a language and environment for statistical computing. R Foundation for Statistical Computing, Vienna, Austria. URL https://www.R-project.org/

[CR33] Ricci A, Li M, Fricke PM, Cabrera VE (2020). Short communication: Economic impact among 7 reproductive programs for lactating dairy cows, including a sensitivity analysis of the cost of hormonal treatments. J Dairy Sci.

[CR34] Routledge EJ, Sumpter JP (1996) Estrogenic activity of surfactants and some of their degradation products assessed using a recombinant yeast screen. Environ Toxicol Chem 15:241–248. 10.1002/etc.5620150303

[CR35] Rovani S, Censi MT, Pedrotti SL, Lima ÉC, Cataluna R, Fernandes AN (2014). Development of a new adsorbent from agro-industrial waste and its potential use in endocrine disruptor compound removal. J Hazard Mater.

[CR36] Sammad A, Umer S, Shi R, Zhu H, Zhao X, Wang Y (2019). Dairy cow reproduction under the influence of heat stress. J Anim Physiol Anim Nutrit.

[CR37] Shrestha SL, Casey FX, Hakk H, Smith DJ, Padmanabhan G (2012). Fate and transformation of an estrogen conjugate and its metabolites in agricultural soils. Environ Sci Technol.

[CR38] Simon E, Duffek A, Stahl C, Frey M, Scheurer M, Tuerk J, Gehrmann L, Könemann S, Swart K, Behnisch P, Olbrich D, Brion F, Ait-Aissa S, Pasanen-Kase R, Werner I, Vermeirssen ELM (2022). Biological effect and chemical monitoring of Watch List substances in European surface waters: steroidal estrogens and diclofenac – effect-based methods for monitoring frameworks. Environ Internat.

[CR39] Wolf Y, Oster S, Shuliakevich A, Brückner I, Dolny R, Linnemann V, Pinnekamp J, Hollert H, Schiwy S (2022). Improvement of wastewater and water quality via a full-scale ozonation plant? – A comprehensive analysis of the endocrine potential using effect-based methods. Sci Total Environ.

[CR40] Yániz JL, Murugavel K, López-Gatius F (2004). Recent developments in oestrous synchronization of postpartum dairy cows with and without ovarian disorders. Reprod Dom Anim.

[CR41] Zhang F, Xie Y, Li X, Wang D, Yang L, Nie Z (2015). Accumulation of steroid hormones in soil and its adjacent aquatic environment from a typical intensive vegetable cultivation of North China. Sci Total Environ.

[CR42] Zhong R, Zou H, Gao J, Wang T, Bu Q, Wang ZL, Hu M, Wang Z (2021). A critical review on the distribution and ecological risk assessment of steroid hormones in the environment in China. Sci Total Environ.

[CR43] Zitnick KK, Shappeli NW, Hakk H, De Sutter TM, Khan E, Casey FXM (2011). Effects of liquid swine manure on dissipation of 17β-estradiol in soil. J Hazard Mater.

