# Correction to: A 4‑year study of bovine reproductive hormones that are induced by pharmaceuticals and appear as steroid estrogenic pollutants in the resulting slurry, using in vitro and instrumental analytical methods

**DOI:** 10.1007/s11356-024-31992-0

**Published:** 2024-01-16

**Authors:** Eduárd Gubó, Judit Plutzer, Tibor Molnár, Dóra Pordán‑Háber, Lili Szabó, Zoltán Szalai, Richard Gubó, Pál Szakál, Tamás Szakál, László Környei, Ákos Bede‑Fazekas, Renátó Kalocsai

**Affiliations:** 1 Albert Kázmér Faculty, Széchenyi István University, Vár tér 2., 9200 Mosonmagyaróvár, Hungary; 2 reAgro Research and Development Ltd., Győrújbarát, Hungary; 3 University of Szeged, Faculty of Science and Informatics, Szeged, Hungary; 4 Geographical Institute, HUN-REN Research Centre for Astronomy and Earth Sciences, Budapest, Hungary; 5 SynCat@Beijing, Synfuels China Technology Co. Ltd., Leyuan South Street II, No.1, Huairou District, Beijing, 101407 China; 6 National Energy Center for Coal to Liquids, Synfuels China Co., Ltd., Beijing, 101400 China; 7 Department of Mathematics and Computational Sciences, Széchenyi István University, Győr, Hungary; 8 Department of Environmental and Landscape Geography, Institute of Geography and Earth Sciences, ELTE Eötvös Loránd University, Budapest, Hungary; 9 MTA Centre of Excellence, HUN-REN CSFK, Budapest, Hungary; 10 HUN-REN Centre for Ecological Research, Institute of Ecology and Botany, Vácrátót, Hungary

**Correction to: Environmental Science and Pollution Research (2023) 30:125596–125608**

10.1007/s11356-023-31126-y

The correct Author affiliations is shown in this paper.

