# Supplementary material for: A 4-year study of bovine reproductive hormones that are induced by pharmaceuticals and appear as steroid estrogenic pollutants in the resulting slurry, using in vitro and instrumental analytical methods
Source: Environ Sci Pollut Res Int. 2023 Nov 25;30(60):125596–608. doi: 10.1007/s11356-023-31126-y (PMC10754748; doi:10.1007/s11356-023-31126-y)
Supplement: Supplementary file 1 — Supplementary file1 (DOCX 150 KB) [file 11356_2023_31126_MOESM1_ESM.docx]

**Supplementary materials**


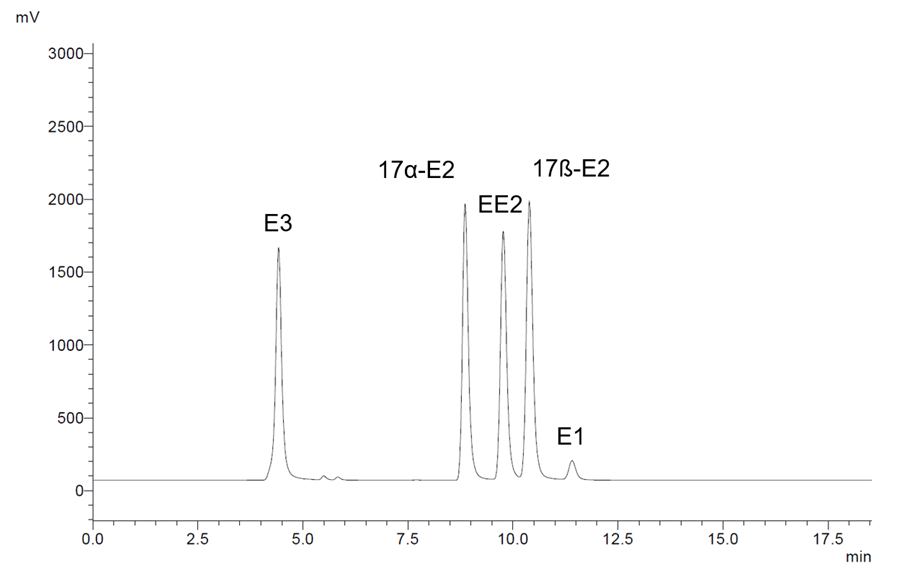


Figure S1.: Chromatograms of estradiols (E1,17α-E2, 17β-E2, EE2, E3) by UHPLC methods


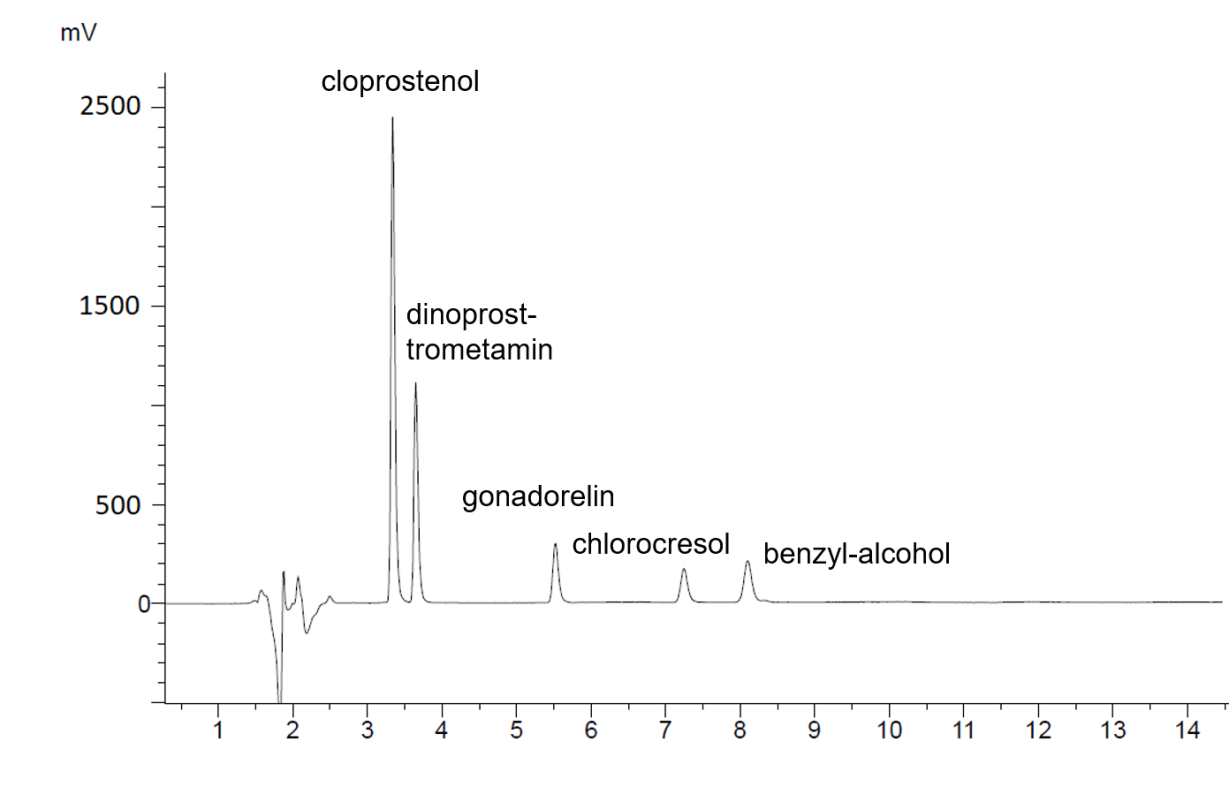


Figure S2.: Chromatograms of main and auxiliary pharmaceuticals (cloprostenol, dinoprost-trometamin, gonadorelin, chlorocresol, benzyl-alcohol) by UHPLC method

Table S1.: Parameters of the UHPLC method (QAQC)

|  |  | PhACs | Concentration (µg/L) | Mean ±SD | Precision (%) | Accuracy (%) |  | Concentration (µg/L) | Mean ±SD | Precision (%) | Accuracy (%) |
| --- | --- | --- | --- | --- | --- | --- | --- | --- | --- | --- | --- |
| Precision | Interday (n=3) | EE2 | 1 | 1.08±0.06 | 5.62 | 8.00 | Intraday (n=3) | 5 | 4.99±0.22 | 4.33 | 0.20 |
|  |  |  | 10 | 9.98±0.32 | 3.21 | 0.20 |  | 50 | 48.93±1.80 | 3.68 | 2.14 |
|  |  |  | 100 | 100.09±0.05 | 0.51 | 0.09 |  | 100 | 99.99±0.78 | 0.78 | 0.01 |
|  |  | 17α-E2 | 1 | 1.09±0.06 | 6.01 | 9.00 |  | 5 | 5.11±0.17 | 3.45 | 2.20 |
|  |  |  | 10 | 10.22±0.22 | 2.14 | 2.20 |  | 50 | 51.93±1.10 | 2.12 | 3.86 |
|  |  |  | 100 | 100.63±0.45 | 0.45 | 0.63 |  | 100 | 100.88±0.55 | 0.55 | 0.88 |
|  |  | 17ß-E2 | 1 | 1.07±0.045 | 4.22 | 7.00 |  | 5 | 5.31±0.17 | 3.12 | 6.20 |
|  |  |  | 10 | 10.45±0.25 | 2.40 | 4.50 |  | 50 | 51.00±0.79 | 1.54 | 2.00 |
|  |  |  | 100 | 99.85±0.22 | 0.22 | 0.15 |  | 100 | 100.32±0.46 | 0.46 | 0.32 |
|  |  | E1 | 1 | 1.07±0.07 | 6.45 | 7.00 |  | 5 | 4.98±0.26 | 5.23 | 0.40 |
|  |  |  | 10 | 9.51±0.12 | 1.23 | 4.90 |  | 50 | 50.89±1.76 | 3.45 | 1.78 |
|  |  |  | 100 | 100.77±0.67 | 0.66 | 0.77 |  | 100 | 99.78±1.00 | 1.00 | 0.22 |
|  |  | E3 | 1 | 1.09±0.06 | 5.41 | 9.00 |  | 5 | 4.63±0.30 | 6.48 | 7.40 |
|  |  |  | 10 | 10.78±0.33 | 3.09 | 7.80 |  | 50 | 49.63±1.11 | 2.23 | 0.74 |
|  |  |  | 100 | 100.35±0.30 | 0.30 | 0.35 |  | 100 | 100.21±0.45 | 0.45 | 0.21 |
|  |  | gonadorelin | 1 | 1.05±0.08 | 7.54 | 5.00 |  | 5 | 4.89±0.25 | 5.20 | 2.20 |
|  |  |  | 10 | 10.45±0.44 | 4.21 | 4.50 |  | 50 | 51.01±1.30 | 2.54 | 2.02 |
|  |  |  | 100 | 100.88±1.11 | 1.10 | 0.88 |  | 100 | 100.32±1.45 | 1.45 | 0.32 |
|  |  | cloprostenol | 1 | 1.09±0.08 | 7.21 | 9.00 |  | 5 | 4.78±0.25 | 5.21 | 4.40 |
|  |  |  | 10 | 10.74±0.29 | 2.66 | 7.40 |  | 50 | 52.01±1.10 | 2.12 | 4.02 |
|  |  |  | 100 | 100.34±1.51 | 1.50 | 0.34 |  | 100 | 101.01±1.46 | 1.45 | 1.01 |
|  |  | dinoprost-trometamin | 1 | 1.09±0.08 | 7.31 | 9.00 |  | 5 | 5.50±0.37 | 6.76 | 10.00 |
|  |  |  | 10 | 10.63±0.26 | 2.45 | 6.30 |  | 50 | 48.35±0.69 | 1.43 | 3.30 |
|  |  |  | 100 | 100.88±1.34 | 1.33 | 0.88 |  | 100 | 100.78±0.68 | 0.67 | 0.78 |
|  |  | chlorocresol | 1 | 1.09±0.15 | 7.99 | 9.00 |  | 5 | 4.89±0.26 | 5.30 | 2.20 |
|  |  |  | 10 | 9.10±0.37 | 4.12 | 9.00 |  | 50 | 53.41±1.19 | 2.24 | 6.82 |
|  |  |  | 100 | 102.41±1.58 | 1.54 | 2.41 |  | 100 | 97.14±2.11 | 2.17 | 2.86 |
|  |  | benzyl-alcohol | 1 | 1.08±0.09 | 8.68 | 8.00 |  | 5 | 5.40±0.38 | 7.10 | 8.00 |
|  |  |  | 10 | 11.01±0.45 | 4.12 | 10.1 |  | 50 | 50.70±1.54 | 3.04 | 1.40 |
|  |  |  | 100 | 101.54±1.43 | 1.41 | 1.54 |  | 100 | 101.60±2.0 | 1.97 | 1.60 |

Table S2. Results of Linearity acquired during validation of the method for analysis of estradiols and pharmaceutical ingredients

| PhACs | Slope | Intercept | R^2^ | Range (µg/mL) |
| --- | --- | --- | --- | --- |
| EE2 | 39724,7 | -244400 | 0,999 | 0,001-2 |
| 17α-E2 | 2909,86 | -26082,7 | 0,999 | 0,001-2 |
| 17ß-E2 | 42970,3 | -220212 | 0,999 | 0,001-2 |
| E1 | 3486,25 | -422,36 | 0,999 | 0,001-2 |
| E3 | 26090,6 | 342265 | 0,999 | 0,001-2 |
| gonadorelin | 5261,19 | -12091,6 | 0,999 | 0,1-1 |
| cloprostenol | 56363,8 | -234003 | 0,999 | 0,1-1 |
| dinoprost-trometamin | 52038,7 | -156100 | 0,999 | 0,1-1 |
| chlorocresol | 5306,6 | -29339,4 | 0,999 | 0,1-1 |
| benzyl-alcohol | 2360,01 | 3052,87 | 0,999 | 0,1-1 |
